# Dibromidobis(3,5-dimethyl-1*H*-pyrazole-κ*N* ^2^)cobalt(II)

**DOI:** 10.1107/S1600536811039560

**Published:** 2011-10-05

**Authors:** Stefania Tomyn, Vadim A. Pavlenko, Elżbieta Gumienna-Kontecka, Larysa Penkova, Natalia V. Kotova

**Affiliations:** a Department of Chemistry, National Taras Shevchenko University of Kyiv, Volodymyrska Street 64, 01601 Kyiv, Ukraine; b University of Wrocław, Faculty of Chemistry, F. Joliot-Curie Street 14, 50-383 Wrocław, Poland

## Abstract

In the mononuclear title complex, [CoBr_2_(C_5_H_8_N_2_)_2_], the Co^II^ atom is coordinated by two N atoms from two monodentate 3,5-dimethyl­pyrazole ligands and two Br atoms in a highly distorted tetra­hedral geometry. In the crystal, the complex mol­ecules are linked by inter­molecular N—H⋯Br hydrogen bonds into chains along [101]. An intra­molecular N—H⋯Br hydrogen bond is also present.

## Related literature

For related structures of pyrazole complexes, see: Krämer & Fritsky (2000 ▶); Sachse *et al.* (2008 ▶); Świątek-Kozłowska *et al.* (2000 ▶); Wörl *et al.* (2005*a* ▶,*b* ▶).
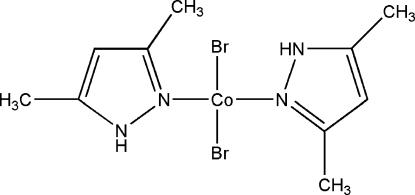


## Experimental

### 

#### Crystal data

[CoBr_2_(C_5_H_8_N_2_)_2_]
*M* *_r_* = 411.00
Monoclinic, 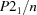

*a* = 8.4729 (4) Å
*b* = 14.1490 (8) Å
*c* = 12.5280 (6) Å
β = 100.152 (4)°
*V* = 1478.38 (13) Å^3^
*Z* = 4
Mo *K*α radiation
μ = 6.55 mm^−1^
*T* = 173 K
0.13 × 0.05 × 0.03 mm

#### Data collection

Oxford Diffraction KM-4 Xcalibur diffractometer
Absorption correction: multi-scan (*CrysAlis RED*; Oxford Diffraction, 2006 ▶) *T*_min_ = 0.420, *T*_max_ = 0.856
16432 measured reflections
4270 independent reflections
3182 reflections with *I* > 2σ(*I*)
*R*_int_ = 0.043

#### Refinement

*R*[*F* ^2^ > 2σ(*F* ^2^)] = 0.035
*wR*(*F* ^2^) = 0.072
*S* = 1.01
4270 reflections
158 parameters
H-atom parameters constrained
Δρ_max_ = 0.95 e Å^−3^
Δρ_min_ = −0.48 e Å^−3^

### 

Data collection: *CrysAlis CCD* (Oxford Diffraction, 2006 ▶); cell refinement: *CrysAlis RED* (Oxford Diffraction, 2006 ▶); data reduction: *CrysAlis RED*; program(s) used to solve structure: *SHELXS97* (Sheldrick, 2008 ▶); program(s) used to refine structure: *SHELXL97* (Sheldrick, 2008 ▶); molecular graphics: *XP* in *SHELXTL* (Sheldrick, 2008 ▶) and *DIAMOND* (Brandenburg, 1999 ▶); software used to prepare material for publication: *SHELXTL*.

## Supplementary Material

Crystal structure: contains datablock(s) global, I. DOI: 10.1107/S1600536811039560/hy2475sup1.cif

Structure factors: contains datablock(s) I. DOI: 10.1107/S1600536811039560/hy2475Isup2.hkl

Additional supplementary materials:  crystallographic information; 3D view; checkCIF report

## Figures and Tables

**Table 1:** Selected bond lengths (Å)

Co1—Br1	2.3841 (4)
Co1—Br2	2.4025 (4)
Co1—N1	2.008 (2)
Co1—N3	2.001 (2)

**Table 2:** Hydrogen-bond geometry (Å, °)

*D*—H⋯*A*	*D*—H	H⋯*A*	*D*⋯*A*	*D*—H⋯*A*
N2—H8⋯Br1	0.80	2.90	3.406 (2)	123
N4—H16⋯Br1^i^	0.89	3.04	3.588 (2)	122

Symmetry code: (i) 
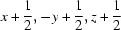
.

**Fig. 1.**
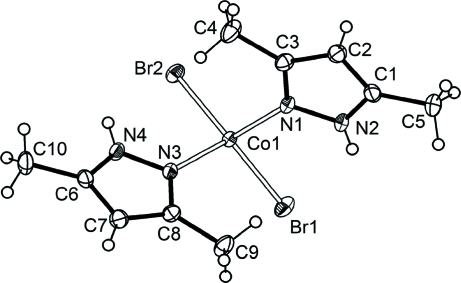
The molecular structure of the title compound. Displacement ellipsoids are drawn at the 50% probability level.

**Fig. 2.**
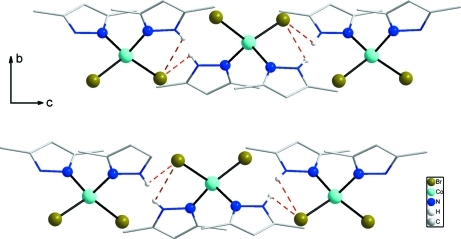
A view of the chain structure. Hydrogen bonds are shown as dashed lines. H atoms not involved in hydrogen bonds are omitted for clarity.

## supplementary crystallographic information

### Comment

In the title mononuclear complex, the Co^II^ atom is coordinated by two N atoms from two monodentate 3,5-dimethylpyrazole ligands and two Br atoms in a highly distorted tetrahedral geometry (Table 1), with the bond angles of 100.49 (6)–119.259 (18)°. The C—C, C—N and N—N bond lengths in the pyrazole ring are normal for 3,5-disubstituted pyrazoles (Krämer & Fritsky, 2000; Sachse *et al.*, 2008; Świątek-Kozłowska *et al.*, 2000; Wörl *et al.*, 2005*a*, *b*). The crystal packing shows a chain-specific arrangement of the molecules. Inside chain intermolecular contacts are ensured by hydrogen bonds between the N—H groups of the pyrazole rings and the bromine atoms (Table 2). Outside chain intermolecular contacts are provided by C—H···Br interactions [H1···Br2^i^ = 2.99 Å, C4—H1···Br2^i^ = 161°; symmetry code: (i) 5/2-x, -1/2+y, 1/2-z].

### Experimental

3,5-Dimethylpyrazole (0.192 g, 2 mmol) was added to a DMSO solution of CoBr_2_.6H_2_O (0.327 g, 1 mmol). The reaction mixture was stirred at 60°C until complete dissolution of the ligand occurred. The resulting blue solution was filtered off and left at room temperature. Block blue crystals suitable for X-ray analysis were isolated by slow evaporation of the resulting solution after several days (yield: 0.35 g, 85%). Analysis, calculated for C_10_H_16_Br_2_CoN_4_: C 29.22, H 3.92, N 13.63%; found: C 29.06, H 3.72, N 13.45%.

### Refinement

The highest positive peak on the residual map was found at 1.38 Å from H8 atom and the deepest hole at 1.05 Å from Br2 atom. H atoms on C atoms were positioned geometrically and refined using a riding model, with C—H = 0.93 (CH) and 0.96 (CH_3_) Å and with *U*_iso_(H) = 1.2(1.5 for methyl)*U*_eq_(C). H atoms on N atoms were located from a difference Fourier map and refined as riding atoms, with *U*_iso_(H) = 1.2*U*_eq_(N).

### Crystal data

**Table:**

[CoBr_2_(C_5_H_8_N_2_)_2_]	*F*(000) = 804
*M**_r_* = 411.00	*D*_x_ = 1.847 Mg m^−^^3^
Monoclinic, *P*2_1_/*n*	Mo *K*α radiation, λ = 0.71073 Å
Hall symbol: -P 2yn	Cell parameters from 20628 reflections
*a* = 8.4729 (4) Å	θ = 3.2–36.6°
*b* = 14.1490 (8) Å	µ = 6.55 mm^−^^1^
*c* = 12.5280 (6) Å	*T* = 173 K
β = 100.152 (4)°	Block, blue
*V* = 1478.38 (13) Å^3^	0.13 × 0.05 × 0.03 mm
*Z* = 4	

### Data collection

**Table:**

Oxford Diffraction KM-4 Xcalibur diffractometer	4270 independent reflections
Radiation source: fine-focus sealed tube	3182 reflections with *I* > 2σ(*I*)
graphite	*R*_int_ = 0.043
ω scans	θ_max_ = 30.0°, θ_min_ = 3.2°
Absorption correction: multi-scan (*CrysAlis RED*; Oxford Diffraction, 2006)	*h* = −11→10
*T*_min_ = 0.420, *T*_max_ = 0.856	*k* = −19→19
16432 measured reflections	*l* = −17→14

### Refinement

**Table:**

Refinement on *F*^2^	Primary atom site location: structure-invariant direct methods
Least-squares matrix: full	Secondary atom site location: difference Fourier map
*R*[*F*^2^ > 2σ(*F*^2^)] = 0.035	Hydrogen site location: difference Fourier map
*wR*(*F*^2^) = 0.072	H-atom parameters constrained
*S* = 1.01	*w* = 1/[σ^2^(*F*_o_^2^) + (0.0341*P*)^2^] where *P* = (*F*_o_^2^ + 2*F*_c_^2^)/3
4270 reflections	(Δ/σ)_max_ = 0.002
158 parameters	Δρ_max_ = 0.95 e Å^−^^3^
0 restraints	Δρ_min_ = −0.48 e Å^−^^3^

### Fractional atomic coordinates and isotropic or equivalent isotropic displacement parameters (Å^2^)

**Table:**

	*x*	*y*	*z*	*U*_iso_*/*U*_eq_	
Br1	0.74035 (3)	0.35425 (2)	0.03635 (2)	0.02328 (8)	
Br2	1.09369 (3)	0.362773 (19)	0.30921 (2)	0.01915 (8)	
Co1	0.91684 (4)	0.27303 (2)	0.17569 (3)	0.01403 (9)	
N1	1.0279 (3)	0.18556 (16)	0.08660 (17)	0.0158 (5)	
N3	0.8064 (3)	0.19278 (16)	0.27161 (17)	0.0161 (5)	
N2	0.9910 (3)	0.18170 (17)	−0.02369 (17)	0.0186 (5)	
H8	0.9180	0.2106	−0.0580	0.022*	
N4	0.8497 (3)	0.19199 (17)	0.38165 (17)	0.0172 (5)	
H16	0.9309	0.2284	0.4122	0.021*	
C3	1.1468 (3)	0.1234 (2)	0.1155 (2)	0.0182 (6)	
C8	0.6852 (3)	0.13054 (19)	0.2490 (2)	0.0169 (5)	
C2	1.1836 (3)	0.0794 (2)	0.0223 (2)	0.0224 (6)	
H4	1.2613	0.0335	0.0197	0.027*	
C7	0.6539 (3)	0.0909 (2)	0.3453 (2)	0.0209 (6)	
H12	0.5757	0.0463	0.3522	0.025*	
C1	1.0810 (3)	0.1181 (2)	−0.0648 (2)	0.0203 (6)	
C6	0.7619 (3)	0.1308 (2)	0.4281 (2)	0.0190 (6)	
C5	1.0621 (4)	0.1005 (3)	−0.1840 (2)	0.0284 (7)	
H5	0.9504	0.0930	−0.2139	0.043*	
H6	1.1189	0.0440	−0.1965	0.043*	
H7	1.1047	0.1531	−0.2182	0.043*	
C10	0.7905 (4)	0.1154 (2)	0.5480 (2)	0.0258 (7)	
H13	0.8020	0.1753	0.5845	0.039*	
H14	0.8865	0.0790	0.5690	0.039*	
H15	0.7012	0.0819	0.5676	0.039*	
C4	1.2209 (4)	0.1101 (2)	0.2318 (2)	0.0282 (7)	
H1	1.2809	0.0522	0.2397	0.042*	
H2	1.1381	0.1074	0.2751	0.042*	
H3	1.2912	0.1621	0.2552	0.042*	
C9	0.6051 (4)	0.1131 (2)	0.1345 (2)	0.0254 (7)	
H9	0.5625	0.0500	0.1282	0.038*	
H10	0.6818	0.1203	0.0871	0.038*	
H11	0.5195	0.1576	0.1147	0.038*	

### Atomic displacement parameters (Å^2^)

**Table:**

	*U*^11^	*U*^22^	*U*^33^	*U*^12^	*U*^13^	*U*^23^
Br1	0.02681 (16)	0.02181 (15)	0.01821 (14)	0.00448 (12)	−0.00429 (11)	0.00067 (11)
Br2	0.02102 (14)	0.01743 (14)	0.01725 (14)	−0.00396 (11)	−0.00142 (11)	−0.00094 (10)
Co1	0.01463 (17)	0.01501 (18)	0.01195 (16)	−0.00021 (15)	0.00096 (12)	−0.00028 (14)
N1	0.0175 (11)	0.0178 (12)	0.0112 (10)	0.0000 (9)	0.0002 (8)	0.0006 (9)
N3	0.0161 (11)	0.0198 (12)	0.0122 (10)	−0.0023 (10)	0.0019 (8)	−0.0010 (9)
N2	0.0195 (12)	0.0251 (13)	0.0107 (10)	0.0011 (10)	0.0014 (9)	0.0005 (9)
N4	0.0184 (11)	0.0202 (12)	0.0121 (10)	−0.0033 (10)	0.0003 (9)	−0.0009 (9)
C3	0.0183 (13)	0.0164 (14)	0.0188 (13)	0.0006 (11)	0.0003 (11)	−0.0028 (11)
C8	0.0172 (13)	0.0154 (13)	0.0175 (12)	−0.0011 (11)	0.0011 (10)	−0.0003 (10)
C2	0.0192 (14)	0.0275 (16)	0.0202 (13)	0.0059 (12)	0.0022 (11)	−0.0062 (12)
C7	0.0199 (14)	0.0224 (15)	0.0203 (14)	−0.0019 (12)	0.0037 (11)	0.0047 (12)
C1	0.0186 (13)	0.0250 (15)	0.0181 (13)	−0.0036 (12)	0.0057 (11)	−0.0046 (11)
C6	0.0185 (13)	0.0231 (15)	0.0163 (12)	0.0023 (12)	0.0055 (10)	0.0036 (11)
C5	0.0294 (17)	0.0402 (19)	0.0157 (14)	−0.0014 (15)	0.0044 (12)	−0.0065 (13)
C10	0.0316 (17)	0.0320 (17)	0.0149 (13)	0.0004 (14)	0.0074 (12)	0.0027 (12)
C4	0.0324 (17)	0.0285 (17)	0.0205 (15)	0.0126 (14)	−0.0041 (12)	−0.0029 (13)
C9	0.0278 (16)	0.0267 (16)	0.0191 (14)	−0.0130 (13)	−0.0030 (12)	0.0014 (12)

### Geometric parameters (Å, °)

**Table:**

Co1—Br1	2.3841 (4)	C2—H4	0.9300
Co1—Br2	2.4025 (4)	C7—C6	1.378 (4)
Co1—N1	2.008 (2)	C7—H12	0.9300
Co1—N3	2.001 (2)	C1—C5	1.495 (4)
N1—C3	1.338 (3)	C6—C10	1.495 (4)
N1—N2	1.363 (3)	C5—H5	0.9600
N3—C8	1.345 (3)	C5—H6	0.9600
N3—N4	1.363 (3)	C5—H7	0.9600
N2—C1	1.339 (4)	C10—H13	0.9600
N2—H8	0.8006	C10—H14	0.9600
N4—C6	1.340 (4)	C10—H15	0.9600
N4—H16	0.8899	C4—H1	0.9600
C3—C2	1.405 (4)	C4—H2	0.9600
C3—C4	1.493 (4)	C4—H3	0.9600
C8—C7	1.398 (4)	C9—H9	0.9600
C8—C9	1.495 (4)	C9—H10	0.9600
C2—C1	1.382 (4)	C9—H11	0.9600
			
N3—Co1—N1	107.38 (9)	N2—C1—C2	106.5 (2)
N3—Co1—Br1	114.45 (6)	N2—C1—C5	121.8 (3)
N1—Co1—Br1	100.66 (6)	C2—C1—C5	131.6 (3)
N3—Co1—Br2	100.49 (6)	N4—C6—C7	106.5 (2)
N1—Co1—Br2	114.62 (6)	N4—C6—C10	121.8 (2)
Br1—Co1—Br2	119.259 (18)	C7—C6—C10	131.7 (3)
C3—N1—N2	106.0 (2)	C1—C5—H5	109.5
C3—N1—Co1	131.23 (18)	C1—C5—H6	109.5
N2—N1—Co1	122.80 (17)	H5—C5—H6	109.5
C8—N3—N4	105.5 (2)	C1—C5—H7	109.5
C8—N3—Co1	131.74 (18)	H5—C5—H7	109.5
N4—N3—Co1	122.75 (17)	H6—C5—H7	109.5
C1—N2—N1	111.8 (2)	C6—C10—H13	109.5
C1—N2—H8	125.1	C6—C10—H14	109.5
N1—N2—H8	122.8	H13—C10—H14	109.5
C6—N4—N3	111.9 (2)	C6—C10—H15	109.5
C6—N4—H16	129.3	H13—C10—H15	109.5
N3—N4—H16	118.7	H14—C10—H15	109.5
N1—C3—C2	109.5 (2)	C3—C4—H1	109.5
N1—C3—C4	120.9 (2)	C3—C4—H2	109.5
C2—C3—C4	129.6 (3)	H1—C4—H2	109.5
N3—C8—C7	109.6 (2)	C3—C4—H3	109.5
N3—C8—C9	120.7 (2)	H1—C4—H3	109.5
C7—C8—C9	129.7 (3)	H2—C4—H3	109.5
C1—C2—C3	106.2 (3)	C8—C9—H9	109.5
C1—C2—H4	126.9	C8—C9—H10	109.5
C3—C2—H4	126.9	H9—C9—H10	109.5
C6—C7—C8	106.5 (3)	C8—C9—H11	109.5
C6—C7—H12	126.8	H9—C9—H11	109.5
C8—C7—H12	126.8	H10—C9—H11	109.5
			
N3—Co1—N1—C3	−63.3 (3)	N2—N1—C3—C4	178.7 (3)
Br1—Co1—N1—C3	176.7 (2)	Co1—N1—C3—C4	−0.2 (4)
Br2—Co1—N1—C3	47.4 (3)	N4—N3—C8—C7	−0.2 (3)
N3—Co1—N1—N2	118.0 (2)	Co1—N3—C8—C7	178.6 (2)
Br1—Co1—N1—N2	−2.1 (2)	N4—N3—C8—C9	179.5 (2)
Br2—Co1—N1—N2	−131.36 (18)	Co1—N3—C8—C9	−1.7 (4)
N1—Co1—N3—C8	−64.7 (3)	N1—C3—C2—C1	0.1 (3)
Br1—Co1—N3—C8	46.1 (3)	C4—C3—C2—C1	−179.2 (3)
Br2—Co1—N3—C8	175.1 (2)	N3—C8—C7—C6	−0.4 (3)
N1—Co1—N3—N4	114.0 (2)	C9—C8—C7—C6	179.9 (3)
Br1—Co1—N3—N4	−135.19 (18)	N1—N2—C1—C2	−1.0 (3)
Br2—Co1—N3—N4	−6.1 (2)	N1—N2—C1—C5	179.6 (3)
C3—N1—N2—C1	1.0 (3)	C3—C2—C1—N2	0.5 (3)
Co1—N1—N2—C1	−179.97 (19)	C3—C2—C1—C5	179.9 (3)
C8—N3—N4—C6	0.9 (3)	N3—N4—C6—C7	−1.1 (3)
Co1—N3—N4—C6	−178.14 (19)	N3—N4—C6—C10	178.2 (2)
N2—N1—C3—C2	−0.6 (3)	C8—C7—C6—N4	0.9 (3)
Co1—N1—C3—C2	−179.5 (2)	C8—C7—C6—C10	−178.3 (3)

### Hydrogen-bond geometry (Å, °)

**Table:**

*D*—H···*A*	*D*—H	H···*A*	*D*···*A*	*D*—H···*A*
N2—H8···Br1	0.80	2.90	3.406 (2)	123
N4—H16···Br1^i^	0.89	3.04	3.588 (2)	122

Symmetry codes: (i) *x*+1/2, −*y*+1/2, *z*+1/2.
